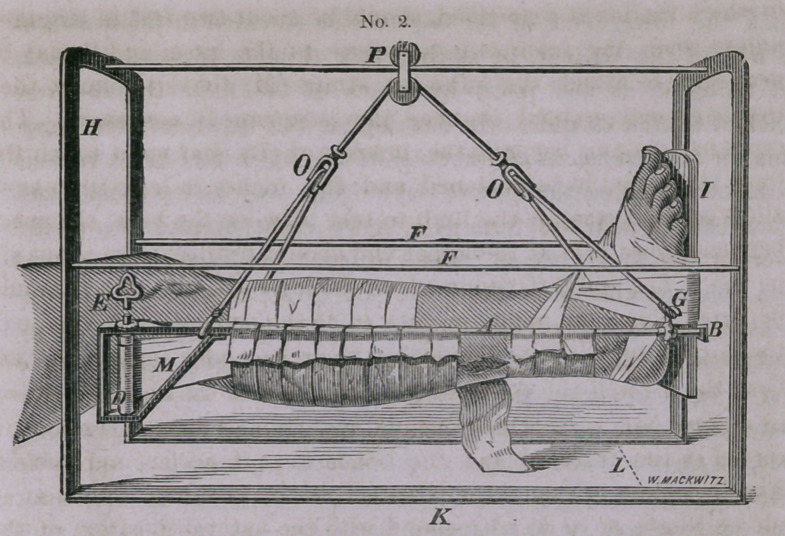# A Suspension Splint, for Treating Simple and Compound Fractures of the Leg

**Published:** 1868-02

**Authors:** E. A. Clark

**Affiliations:** Resident Physician, St. Louis City Hospital


### Miscellaneous.

### A Suspension Splint, for treating Simple and Compound Fractures of the Leg.

By E. A. CLARK, M. D., Resident Physician, St. Louis City Hospital.

The great necessity for a well adapted apparatus in treating fractures of the leg, suggested the utility of the instrument I have designed in the following wood-cut, which, not only answers every practical purpose in treating this class of fractures, but also contributes very much to the comfort of the patient, who, while he is enabled to execute every movement of which the sound limb is capable, yet, cannot displace the fracture or modify the force of extension. In presenting this apparatus, I claim an advantage over those invented by Hutchinson, John Neill, Crandall and Salter, not only for the means of extension and counter-extension, but also its adaptation to the treatment of compound fractures of the leg, as represented in figure No. 2. And considering the simplicity of this instrument, with its cheapness and application to every variety of fractures of the leg, will certainly give it the precedence with those who may venture to use it in a single case. The apparatus is such as may be made by any blacksmith, or indeed by any ingenious surgeon in a case of necessity, when a wooden frame and two hoops with a common iron pully will answer quite as well as the instrument which I have had made of iron on the following plan:

No. 1.

The two arches represented by the letter (H), at one end, are made of iron bars one-eighth of an inch in thickness, and three-fourths of an inch wide. These arches are continuous with the bottom pieces (K), which support them upon the bed and measure twenty-two inches in length, making the distance between the two arches, which are also supported on the sides by the two slender bars (F. F.) While the bar extending across the top, upon which the pully (P) glides, should be made flat, with the long diameter perpendicular so as to prevent it bending with the weight of the leg. The width of the arch under which the leg is suspended—as indicated by the letter (L), should be fifteen inches, and the arch eighteen inches from the surface of the bed.

This description will be sufficient to indicate the proportions of the exterior apparatus. The bars represented by the letter (A), in which the leg is suspended, should be about two feet in length—unless when the fracture is too close to the knee, and it may be necessary to attach the adhesive straps (M) above the knee, then the bars may extend to near the perineum if necessary. The cross-bar passing beneath the bracket at (B), and upon which the foot rests, should be flattened and five inches in length, so as to allow ample space for the limb to rest between the bars; the space between these bars at the upper end should ordinarily be about six inches. The splint (C) upon which the leg rests in figure 1, should be fluted upon its upper surface so as to conform to the shape of the leg, while it is also made oval upon its under surface, so that both the leg and the splint may be included in the bandage shown in figure No. 1, by which means any displacement may be corrected in the fracture and the bones kept in perfect apposition. The foot piece (1) should be attached to the posterior splint at an obtuse angle, so as to correspond with the natural position of the foot. The foot is bound to this piece by means of adhesive straps which may embrace the whole of the foot, and extend partly over the ankle, but not so as to arrest the circulation, as by the figure of eight bandage used around the ankle for making extension. The leg then, as seen in figure No. 1, is supported upon the cross-bár passing under the bracket (B) attached to the foot-piece, and by resting upon the strap (N), pinned over the bars (A) on either side; while the extension and counter-extension is effected by means of the bar across the foot-piece below, and above by means of adhesive straps three inches in width, as indicated by the letter (M), which are attached to the sides of the leg, beginning just above the point of fracture and passing up to the wound around the cylinder (D), which is three and a half inches in length, and turned by means of an ordinary clock key, represented by the letter (E.) This cylinder is held in any position to which it may be turned, by a ratchet and wheel placed upon the upper surface of the bar, as indicated in the diagram.

No. 2.

It will be observed in figure No. 2, that there is no posterior splint as in the other diagram, but the leg is supported entirely by strips of muslin pinned over the bars on either side, which renders this apparatus more appropriate for the treatment of compound fractures in which the wound may be examined and dressed when necessary, by removing one or more of these strips which may be replaced by new ones without disturbing the fracture. The attachment of the foot-piece in this dressing does not in any particular differ from that of figure No. 1. The means of suspension is the same in both these dressings, which, by means of the pully at the letter (P), the patient is enabled to move his limb, or even his body, forward and back to the extent of the length of the bar upon which it glides, and by means of the cord playing over the under wheel in the same pully, the patient is able to flex and extend the knee by depressing or elevating the foot, which movement can be executed by a very slight effort on the part of the patient, while at the same time he can swing the leg from side to side to any extent within the space of the arches; and by means of the cords playing through the pulleys at (O. O.), the leg can be rotated to any extent, even to allow the patient to lie upon his side if he desires, without disturbing the fracture in the least. It will be observed in the diagrams that at the letter (G) there is a thimble, which can be made to slide upon the bar, by means of which the lower end of the leg can be elevated or depressed at the will of the patient, by sliding this thimble forward or back, and fixing it at any point by means of the little thumb-screw attached to this thimble. In developing the utility of this apparatus for the treatment of fractures of the leg, I have tried various means of attaching the foot at the bottom, such as the muslin and flannel bandages in the form of a figure eight around the ankle, covering the foot also, as far as the toes; but have always found them objectionable from the great amount of pressure and consequent arrest of the circulation in the foot, though the flannel bandage is much less objectionable than the muslin. But I have been able to obviate this objection, by the use of the adhesive plaster attached over the front of the foot, and around the foot-piece, as shown in the diagram; this I have ordinarily found quite sufficient, unless in rare cases, when an unusual counter-extending force is required, it may become necessary—as very aptly suggested by Prof. Hammer of this city—to pass a strip of adhesive plaster beneath the heel and around the foot-piece, which adds very much to the strength of the dressing. I have recently treated six cases of fractures of the leg with this apparatus, in which both bones were fractured, and in which there was more or less shortening in each case, with excellent results in all of them, without allowing the least deformity or shortening, while the patients were all grateful for the comforts allowed them by this apparatus during their confinement.—*Humboldt Medical Archives.*

## Figures and Tables

**No. 1. f1:**
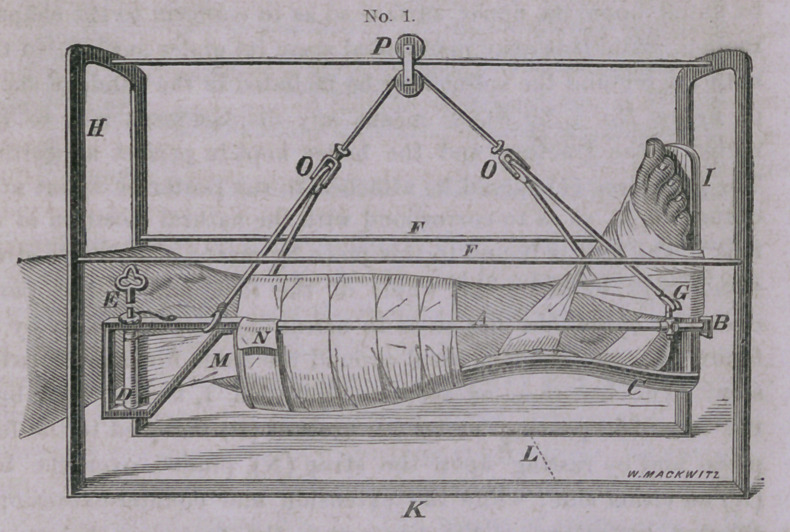


**No. 2. f2:**